# Supplementary material for: Genomics knowledge and attitudes among European public health professionals: Results of a cross-sectional survey
Source: PLoS One. 2020 Apr 2;15(4):e0230749. doi: 10.1371/journal.pone.0230749 (PMC7117699; doi:10.1371/journal.pone.0230749)
Supplement: S1 Text — (DOC) [file pone.0230749.s001.doc]

**S1.TEXT**

**QUESTIONNAIRE ON KNOWLEDGE AND ATTITUDES ON PUBLIC HEALTH GENOMICS**

**A. PERSONAL DETAILS**

*In this section we ask you to provide some personal details.*

**A1. Birth Year** __________________________________

**A2. Gender**

- F
- M

**A3. In which Country do you conduct your professional activity?**

___________________________________________

**A4. Do you have a personal or family history of a genetic disorder or hereditary syndrome?**

- Yes
- No

**B. PROFESSIONAL ACTIVITY**

*In this section we ask you to provide some information on your education and your professional activities.*

**B1. Which kind of health professional are you?**

- I am a Public Health Professional not involved in Public Health Genomics (PHG)
- I am a Public Health Professional involved in PHG activities
- I am not a Public Health Professional, and I am not involved in PHG
- I am not a Public Health professional, but I am involved in PHG

*According to WHO definition “public health professionals monitor and diagnose the health concerns of entire communities and promote healthy practices and behaviours to ensure that populations stay healthy”.*

*Public Health Genomics (PHG) is defined as “the responsible and effective translation of genome-based knowledge and technologies into public policy and health services for the benefit of population health”. The term is not used consistently throughout Europe, where some countries (e.g. the Netherlands) refer to this specific field as “community genetics”. Practical examples of PHG are the introduction of genomic testing into cancer screening programmes and the use of genetic information for the prevention of cardiovascular diseases.*

**B2. What is the highest educational degree obtained?**

- Bachelor‘s
- Master’s
- Doctorate
- Other (please specify)_________________________

**B3. Area of degree**

- Medicine
- Health professions (nursing, etc.)
- Biology
- Psychology
- Statistics
- Economics
- Other or mixes (please specify)_________________________

**B4. Has information on genetic testing been addressed during your undergraduate training?**

- Yes
- No

**B5. Has information on genetic testing been addressed during your postgraduate training?**

- Yes
- No
- Not applicable

**B6. What sector do you work in?**

- Academic/research
- Hospital
- Technical Agency
- National Government (i.e. Ministry of Health)
- Local Government (i.e. Regional Health Authorities)
- Public health service (e.g. vaccination service/screening program/maternal-child health service, etc.)
- Other (please specify)_________________________

**B7. What are your main areas of work? (maximum 3 answers** are allowed)

| - Statistics and epidemiology | - Public health policy |
| --- | --- |
| - Health economics | - Public health genomics |
| - Communicable diseases control | - Health technology assessment |
| - Non-communicable diseases control | - Health impact assessment |
| - Environment related diseases control | - Migrant and ethnic minorities health |
| - Food and nutrition | - Public mental health |
| - Health services management | - [Injury prevention and safety promotion](http://www.eupha.org/injury-prevention-and-safety-promotion) |
| - Health services research | - Child and adolescent health |
| - Others (please specify)_________________ | |

**C. KNOWLEDGE ON GENETIC TESTING AND THE DELIVERY OF GENETIC SERVICES**

*In this section we ask about your knowledge on genetic testing and the delivery of genetic services.*

##### C1. Which of the following applications of genetic testing are based on evidence of effectiveness? (multiple answers are possible)

| - Diagnose disease | - Identify genetic mutations that influence the process of ageing |
| --- | --- |
| - Determine the severity of a disease | - Guide doctors in deciding on the best treatment to use for certain individuals |
| - Identify genetic mutations that are responsible for an already diagnosed disease | - Guide doctors in designing an optimal individualized weight loss diet |
| - Identify genetic mutations that may increase the risk to develop a disease | - Ascertain the gender of a fetus |
| - Identify genetic mutations that could be passed on to children | - Screen newborn babies for certain treatable conditions |

**C2. Recommendations/guidelines produced by authoritative health organizations about the use of susceptibility (or predisposition) tests already exist.[[1]](#footnote-2)**

- Yes
- No
- I don’t’ know

**C3. For how many clinical conditions is there currently a base of synthesized evidence supporting the implementation of genetic testing into practice to predict disease risk? [[2]](#footnote-3)***

- None
- <15
- 15-50
- 51-100
- >100

**C4. For which of the following clinical conditions is there currently a base of synthesized evidence supporting the implementation of genetic testing to predict individual risk of disease? (multiple answers** are possible)

| - Hereditary ovarian cancer | - Alzheimer disease |
| --- | --- |
| - Lynch syndrome (hereditary nonpolyposis colorectal cancer) | - Familial hypercholesterolemia |
| - Gastric cancer | - Type 2 diabetes |
| - Metastatic non-small-cell lung cancer | - Acute myeloid leukemia |
| - Prostate cancer | - Depression |

**C5**. **Which of the following professionals may be involved in the delivery of genetic testing?**

1. General practitioner
2. Geneticist
3. Oncologist
4. All of the above
5. B+C

**C6. Which should be the components of a genetic service? (multiple answers are possible)[[3]](#footnote-4)***

1. Genetic testing
2. Diagnosis
3. Collection of family health history and risk assessment
4. Counselling
5. Treatment for individuals with, or at risk of, genetic disorders
6. Follow-up of patients
7. Clinical surveillance of individuals with, or at risk of, genetic disorders

**C7. Performing a susceptibility (or predisposition) test should necessarily be associated with genetic counseling that includes information, informed consent, and discussion of the results. ***

- Yes
- No
- I don’t’ know

**C8. A standardized set of process and outcome indicators to evaluate genetic services, such as those existing to evaluate maternal health services (e.g. n. of health centers providing essential obstetric care/500,000 population; n. of deliveries/place of birth; neonatal mortality/place of birth) already exist.^*^**

- Yes
- No
- I don’t know

**D. ATTITUDES ON GENETIC TESTING AND THE DELIVERY OF GENETIC SERVICES**

*In this section we ask your opinion on some statements regarding genetic testing and the delivery of genetic services.*

**D1. It is more important to invest resources in the social and environmental causes of ill health than in the implementation of genetic testing.**

- Strongly agree
- Agree
- Neither agree nor disagree
- Disagree
- Strongly disagree

**D2.** **Susceptibility (or predisposition) tests should be introduced in the clinical and public health practice even without health interventions with proven efficacy.**

- Strongly agree
- Agree
- Neither agree nor disagree
- Disagree
- Strongly disagree

**D3. Susceptibility (or predisposition) tests should be introduced in the clinical and public health practice only if economic evaluations show cost-effectiveness ratios favorable compared with alternative health interventions.**

- Strongly agree
- Agree
- Neither agree nor disagree
- Disagree
- Strongly disagree

**D4. The application of genetic testing in healthy subjects may increase prevention opportunities for chronic diseases, such as hereditary cancer and hereditary cardiovascular disease.[[4]](#footnote-5)***

- Strongly agree
- Agree
- Neither agree nor disagree
- Disagree
- Strongly disagree

**D5. Equal accessibility and effectiveness of genetic services can be facilitated by integrating them into the already available health services (e.g. hospital cancer units, GP practices, public health programs). [[5]](#footnote-6)***

- Strongly agree
- Agree
- Neither agree nor disagree
- Disagree
- Strongly disagree

**D6. Regulatory approaches are needed to control the direct-to-consumer market industry in genetic testing. ^*^**

- Strongly agree
- Agree
- Neither agree nor disagree
- Disagree
- Strongly disagree

**D7. Genetic tests for diseases that could have a fatal outcome (e.g. BRCA testing for breast and ovarian cancer) should be provided free at the point of delivery (by the healthcare system) to people who could benefit from them.**

- Strongly agree
- Agree
- Neither agree nor disagree
- Disagree
- Strongly disagree

**D8. Specific training initiatives are needed for public health professionals to develop their capacity to design and evaluate the quality of genetic services. ^*^**

- Strongly agree
- Agree
- Neither agree nor disagree
- Disagree
- Strongly disagree

**E. ATTITUDES REGARDING THE ROLES OF PUBLIC HEALTH PROFESSIONALS IN PHG**

*Following the completion of the human genome project in 2003 a debate has arisen on the possible utility of genomic science for public health purposes. Some public health professionals considered a limited role of genomics in public health, which should instead focus on the environmental causes of disease (i.e., infectious, chemical, behavioral, and social factors).* *On the other hand, several public health practitioners have supported the incorporation of genome-based knowledge and technologies into public health.*

*The following statements are based on published literature dealing with the relationship between public health and human genomics and the possible role of public health professionals in putting PHG into practice. To what extent do you agree with each of these statements?*

**E1. Public health thinking should consider that risk factors can affect subsets of the population differently based on genetic susceptibility.***

- Strongly agree
- Agree
- Neither agree nor disagree
- Disagree
- Strongly disagree

**E2. Public health professionals should be involved in the continuous assessment of the utility and validity of emerging genomic applications.***

- Strongly agree
- Agree
- Neither agree nor disagree
- Disagree
- Strongly disagree

**E3. Public health programs should actively implement genomic applications that are evidence-based (e.g. BRCA testing for relatives of known mutation carriers).***

- Strongly agree
- Agree
- Neither agree nor disagree
- Disagree
- Strongly disagree

**E4. Public health professionals should measure the utilization of genetic services in order to assess unmet needs and inequalities of access to services.***

- Strongly agree
- Agree
- Neither agree nor disagree
- Disagree
- Strongly disagree

**E5. Public health professionals should measure in practice outcomes, process indicators and value added of genomic applications.***

- Strongly agree
- Agree
- Neither agree nor disagree
- Disagree
- Strongly disagree

**E6. I think that in the future public health programmes (e.g. cancer screening programmes, chronic diseases prevention programmes) will make a stronger use of genetic information.***

- Strongly agree
- Agree
- Neither agree nor disagree
- Disagree
- Strongly disagree

***Genetic testing***: performing a type of medical test involving an analysis of human chromosomes, DNA, RNA, genes, and/or gene products (e.g., enzymes and other types of proteins), which is predominately used to detect heritable or somatic mutations, genotypes, or phenotypes related to disease and health.

***Susceptibility testing*** ***(also known as predisposition test)*** detects genetic variants that are associated with an increased risk of disease but cannot predict with certainty the development of disease, because of the incomplete penetrance of the genetic mutation. It differs from predictive genetic test (also known as pre-symptomatic test), which is the use of genetic testing to predict whether an individual will develop a genetic disease at a later stage of their life: this term is only applicable where the disease-associated mutation is known and highly penetrant.

*NB: for the scope of this survey, we decided to adopt the classification of genetic tests based on “Stewart A et al. Genetics, Health Care and Public Policy. An Introduction to Public Health Genetics. Cambridge: Cambridge University Press, 2007. pp 108-9”. However, other classifications exists, such as that proposed by the US National Institute of Health’s Genetic Home Reference (see* [*http://ghr.nlm.nih.gov/handbook/testing/uses*](http://ghr.nlm.nih.gov/handbook/testing/uses)*), where “Predictive tests” are defined as genetic tests that can identify mutations that increase a person's chances of developing disorders with a genetic basis, differing from “presymptomatic testing”, which can determine whether a person will develop a genetic disorder before any signs or symptoms*

***Direct-to-consumer genetic test***: genetic tests that are both marketed and sold directly

to the public, including over the counter, without the supervision of a healthcare professional

1. Only professionals involved in PHG activities answered to this question [↑](#footnote-ref-2)
2. ^*^ [↑](#footnote-ref-3)
3. ^*^ Only professionals involved in PHG activities answered to this question [↑](#footnote-ref-4)
4. ^*^ Only professionals involved in PHG activities answered to this question [↑](#footnote-ref-5)
5. ^*^ Only professionals involved in PHG activities answered to this question [↑](#footnote-ref-6)
