# Supplementary material for: Genomics knowledge and attitudes among European public health professionals: Results of a cross-sectional survey
Source: PLoS One. 2020 Apr 2;15(4):e0230749. doi: 10.1371/journal.pone.0230749 (PMC7117699; doi:10.1371/journal.pone.0230749)
Supplement: S1 Table — (DOCX) [file pone.0230749.s003.docx]

**Table 2_bis Knowledge on genetic testing and the delivery of genetic services, n(%)**

| *Which of the following applications of genetic testing are based on evidence of effectiveness? (multiple answers are possible) (n=93)*^a^* | | No | | Yes |
| --- | --- | --- | --- | --- |
| Diagnose disease | | 40 (43.0) | | **53 (57.0)** |
| Determine the severity of a disease | | **77 (82.8)** | | 16 (17.2) |
| Identify genetic mutations that are responsible for an already diagnosed disease | | 28 (30.1) | | **65 (69.9)** |
| Identify genetic mutations that may increase the risk to develop a disease | | 27 (29.0) | | **66 (71.0)** |
| Identify genetic mutations that could be passed on to children | | 31 (33.3) | | **62 (66.7)** |
| Identify genetic mutations that influence the process of ageing | | **72 (77.4)** | | 21 (22.6) |
| Guide doctors in deciding on the best treatment to use for certain individuals | | 42 (45.2) | | **51 (54.8)** |
| Guide doctors in designing an optimal individualized weight loss diet | | **85 (91.4)** | | 8 (8.6) |
| Ascertain the gender of a fetus | | **61 (65.6)** | | 32 (34.4) |
| Screen newborn babies for certain treatable conditions | | 30 (32.3) | | **63 (67.7)** |
| *Recommendations/guidelines produced by authoritative health organizations about the use of susceptibility (or predisposition) tests already exist (n=50)* | | | | |
| Agree | |  | | **38 (76.0)** |
| Uncertain | |  | | 9 (18.0) |
| Disagree | |  | | 3 (6.0) |
| *For how many clinical conditions is there currently a base of synthesized evidence supporting the implementation of genetic testing into practice to predict disease risk (n=33)* | | | | |
| None | |  | | 4 (12.1) |
| <15 | |  | | **4 (12.1)** |
| 15-50 | |  | | 15 (45.4) |
| 51-100 | |  | | 5 (15.2) |
| >100 | |  | | 5 (15.2) |
| *For which of the following clinical conditions is there currently a base of synthesized evidence supporting the implementation of genetic testing to predict disease risk? (multiple answers are possible) (n=93)*^b^* | | No | | Yes |
| Hereditary ovarian cancer | | 27 (29.0) | | **66 (71.0)** |
| Lynch syndrome (hereditary nonpolyposis colorectal cancer) | | 42 (45.2) | | **51 (54.8)** |
| Gastric cancer | | **80 (86.0)** | | 13 (14.0) |
| Metastatic non-small-cell lung cancer | | **82 (88.2)** | | 11 (11.8) |
| Prostate cancer | | **81 (87.1)** | | 12 (12.9) |
| Alzheimer disease | | **74 (79.6)** | | 19 (20.4) |
| Familial hypercholesterolemia | | 41 (44.1) | | **52 (55.9)** |
| Type 2 diabetes | | **78 (83.9)** | | 15 (16.1) |
| Acute myeloid leukemia | | **78 (83.9)** | | 15 (16.1) |
| Depression | | **82 (88.2)** | | 11 (11.8) |
| *Which of the following professionals may be involved in the delivery of genetic testing?(n=89)** | | | | |
| 1. General practitioner | | | | 1 (1.1) |
| 1. Geneticist | | | | 8 (9.0) |
| 1. Oncologist | | | | - - |
| 1. All of the above | | | | **55 (61.8)** |
| 1. B+C | | | | 25 (28.1) |
| *Which should be the components of a genetic service? (multiple answers are possible) (n=53)* | No | | | Yes |
| Genetic testing | 6 (11.3) | | | **47 (88.7)** |
| Diagnosis | 22 (41.5) | | | **31 (58.5)** |
| Collection of family health history and risk assessment | 9 (17.0) | | | **44 (83.0)** |
| Counselling | 10 (18.9) | | | **43 (81.1)** |
| Treatment for individuals with, or at risk of, genetic disorders | 33 (62.3) | | | **20 (37.7)** |
| Follow-up of patients | 27 (50.1) | | | **26 (49.1)** |
| Clinical surveillance of individuals with, or at risk of, genetic disorders | 28 (52.8) | | | **25 (47.2)** |
| *Performing susceptibility (or predisposition) tests should necessarily be associated with genetic counseling that includes information, informed consent, and discussion of the results (n=88)** | | | | |
| Agree | | | | **79 (89.8)** |
| Uncertain | | | | 4 (4.5) |
| Disagree | | | | 5 (5.7) |
| *A standardized set of process and outcome indicators to evaluate genetic services, such as those existing to evaluate maternal health services (e.g. n. of health centers providing essential obstetric care/500,000 population; n. of deliveries/place of birth; neonatal mortality/place of birth) already exist(n=49)* | | | | |
| Agree | | | 13 (26.5) | |
| Uncertain | | | 13 (26.5) | |
| Disagree | | | **23 (47.0)** | |

*Number of respondents to the question

^a^A correct response to the question was defined as having correctly identified at least 8/10 applications of genetic testing that are based (or not) on evidence of effectiveness

^b^A correct response to the question was defined as having correctly identified all conditions for which there is currently (or there is not) evidence of effectiveness for the implementation of genetic testing

Note: Percentages referring to correct answers are in bold.

**Table 3_bis. Attitudes on genetic testing and the delivery of genetic services (% of answers)**

| Statement | Strongly agree | Agree | Neither agree nor disagree | Disagree | Strongly disagree |
| --- | --- | --- | --- | --- | --- |
| *It is more important to invest resources in the social and environmental causes of ill health than in the implementation of genetic testing (n=88)** | 18.2 | 22.7 | 31.8 | **26.1** | **1.1** |
| *Susceptibility (or predisposition) tests should be introduced in the clinical and public health practice even without health interventions with proven efficacy (n=88)** | 3.4 | 12.5 | 7.9 | **45.5** | **30.7** |
| *Susceptibility (or predisposition) tests should be introduced in the clinical and public health practice only if economic evaluations show cost-effectiveness ratios favorable compared with alternative health interventions (n=88)** | **19.3** | **38.6** | 17.1 | 19.3 | 5.7 |
| *The application of genetic testing in healthy subjects may increase prevention opportunities for chronic diseases, such as hereditary cancer and hereditary cardiovascular disease (n=49)* | **24.5** | **53.1** | 12.2 | 6.1 | 4.1 |
| *Equal accessibility and effectiveness of genetic services can be facilitated by integrating them into the already available health services (e.g. hospital cancer units, GP practices, public health programs (n=49)* | **34.7** | **57.1** | 6.1 | 2.1 | - |
| *Regulatory approaches are needed to control the direct-to-consumer market industry in genetic testing (n=49)* | **65.3** | **28.6** | 6.1 | - | - |
| *Genetic tests for diseases who could have a fatal outcome (e.g. BRCA testing for breast and ovarian cancer) should be provided free at the point of delivery to people who could benefit from them (n=88)** | **34.1** | **41.0** | 12.5 | 7.9 | 4.5 |
| *Specific training initiatives are needed for public health professionals to develop their capacity to design and evaluate the quality of genetic services (n=49)* | **28.3** | **43.7** | 11.5 | - | - |

*Number of respondents to the question

Note: Percentages referring to positive attitudes towards genetic testing and delivery of genetic services are in bold.
